# A Comparative Analysis of the Mamba, Transformer, and CNN Architectures for Multi-Label Chest X-Ray Anomaly Detection in the NIH ChestX-Ray14 Dataset

**DOI:** 10.3390/diagnostics15172215

**Published:** 2025-09-01

**Authors:** Erdem Yanar, Furkan Kutan, Kubilay Ayturan, Uğurhan Kutbay, Oktay Algın, Fırat Hardalaç, Ahmet Muhteşem Ağıldere

**Affiliations:** 1 Department of Healthcare Systems System Engineering, ASELSAN, 06200 Ankara, Turkey; 2 Department of Test and Verification Engineering, ASELSAN, 06200 Ankara, Turkey; furkan.kutan@gazi.edu.tr; 3 Department of Electrical and Electronics Engineering, Gazi University, 06570 Ankara, Turkey; kubilay.ayturan@gazi.edu.tr (K.A.); ukutbay@gazi.edu.tr (U.K.);; 4 Department of Radiology, Medical Faculty, Ankara University, 06230 Ankara, Turkey; oktay.algin@umram.bilkent.edu.tr; 5 Department of Radiology, Faculty of Medicine, Baskent University, 06490 Ankara, Turkey; muhtesema@gmail.com

**Keywords:** chest X-ray, thoracic disease detection, multi-label classification, transformer architectures, mamba architecture, NIH ChestX-ray14, AUROC evaluation, diagnostic decision support

## Abstract

**Background/Objectives:** Recent state-of-the-art advances in deep learning have significantly improved diagnostic accuracy in medical imaging, particularly in chest radiograph (CXR) analysis. Motivated by these developments, a comprehensive comparison was conducted to investigate how architectural choices affect performance of 14 deep learning models across Convolutional Neural Networks (CNNs), Transformer-based models, and Mamba-based State Space Models. **Methods:** These models were trained and evaluated under identical conditions on the NIH ChestX-ray14 dataset, a large-scale and widely used benchmark comprising 112,120 labeled CXR images with 14 thoracic disease categories. **Results:** It was found that recent hybrid architectures—particularly ConvFormer, CaFormer, and EfficientNet—deliver superior performance in both common and rare pathologies. ConvFormer achieved the highest mean AUROC of 0.841 when averaged across all 14 thoracic disease classes, closely followed by EfficientNet and CaFormer. Notably, AUROC scores of 0.94 for hernia, 0.91 for cardiomegaly, and 0.88 for edema and effusion were achieved by the proposed models, surpassing previously reported benchmarks. **Conclusions:** These results not only highlight the continued strength of CNNs but also demonstrate the growing potential of Transformer-based architectures in medical image analysis. This work contributes to the literature by providing a unified, state-of-the-art benchmarking of diverse deep learning models, offering valuable guidance for researchers and practitioners developing clinically robust AI systems for radiology.

## 1. Introduction

Chest radiography (CXR) continues to be one of the most frequently used diagnostic modalities in medicine due to its accessibility, rapid image acquisition, and cost-effectiveness [1]. Its utility spans a broad range of clinical scenarios, from emergency triage and infection detection to chronic disease surveillance and cancer screening. As a first-line imaging tool, CXR plays a pivotal role in detecting thoracic abnormalities such as pneumonia [2], cardiomegaly [3], pleural effusion, and pulmonary nodules, especially in settings with limited access to advanced imaging modalities like CT or MRI [4].

Despite its widespread use, manual interpretation of CXRs remains challenging. Radiologists must interpret subtle grayscale variations across overlapping anatomical structures, often under significant time pressure. Studies have shown that this complexity leads to inter-observer variability, with kappa values as low as 0.2–0.6 for many pathologies, including pneumothorax, pneumonia, and consolidation [5]. Additionally, radiographic signs of different conditions (e.g., atelectasis vs. pneumonia vs. consolidation) may visually resemble each other, making it difficult to distinguish between etiologies without supplementary clinical information. As healthcare systems confront growing image volumes, diagnostic complexity, and workforce shortages, the need for automated, accurate, and efficient AI systems to support CXR interpretation has never been more urgent.

In recent years, deep learning (DL) architectures—especially Convolutional Neural Networks (CNNs) and Vision Transformers (ViTs)—have made significant advances in CXR interpretation tasks [6]. Pioneering models like CheXNet (DenseNet121) have demonstrated performance comparable to radiologists for pneumonia detection in the NIH ChestX-ray14 dataset, while more recent Transformer-based models, including architectures like SwinCheX, have shown improved performance in multi-label thoracic anomaly detection [7]. More recently, State Space Models like Mamba variants have been proposed to efficiently capture long-range dependencies with reduced computational cost, offering a promising alternative to ViTs. However, these architectures still face challenges: CNNs often struggle to capture long-range spatial relationships and can be sensitive to visual noise, ViTs are limited by quadratic self-attention complexity in high-resolution images, and Mamba-based models require further optimization to fully realize their potential in medical imaging tasks.

The NIH ChestX-ray14 dataset [176 source] presents significant challenges due to its complexity and size. It includes over 112,000 frontal chest X-ray images (CXRs), each labeled for 14 common thoracic diseases. Some of these diseases, like hernia (0.20% prevalence), fibrosis (1.50%), and pneumonia (1.28%), are quite rare but clinically important. This class imbalance—where some diseases appear much less frequently than others—makes training models difficult because they tend to focus on more common conditions such as infiltration (17.74%) and effusion (11.88%), often at the expense of accurately detecting the less frequent but critical diseases. The distribution of these 14 pathologies within the dataset is illustrated in Table 1.

**Figure 1:**
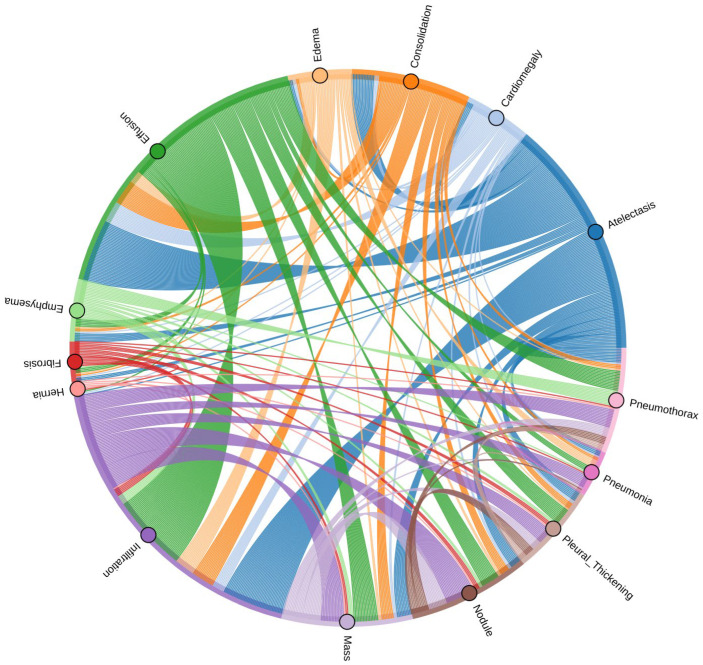
Chord diagram illustrating co-occurrence relationships among the 14 thoracic diseases in the ChestX-ray14 dataset.

Furthermore, many chest X-rays in the dataset exhibit multiple diseases simultaneously, leading to significant label co-occurrence and challenging the common assumption of label independence in multi-label classification tasks. For instance, effusion and cardiomegaly often appear together in patients with congestive heart failure, while emphysema and pneumothorax are frequently co-diagnosed during COPD exacerbations [8]. This high degree of co-occurrence introduces additional complexity to both model training and evaluation, as models must learn to recognize overlapping patterns associated with different conditions. The detailed distribution of the 14 pathologies, along with their co-occurrence relationships, is visually depicted using a chord diagram, which highlights common disease pairings, in Figure 1.

Automated chest X-ray interpretation faces several key challenges, including overlapping anatomical structures, class imbalance, and the presence of multiple labels occurring together. These issues make it difficult for models to accurately detect thoracic abnormalities, especially when working with weakly supervised medical datasets that contain label noise and overlapping conditions. In this study, these challenges are addressed through a thorough benchmarking of modern deep learning architectures for multi-label thoracic anomaly detection.

To achieve this, three main families of models are explored: Convolutional Neural Networks (CNNs), Transformer-based models, and the newer State Space Model (SSM)-based Mamba variants. The CNN models included in this study are DenseNet121, ResNet34, InceptionV3, and EfficientNet, which are widely used in medical imaging tasks. The Transformer models evaluated in this study are ConvFormer, CaFormer, Swin Transformer, and DeiT, all known for their strong performance. Additionally, Mamba-based models—MedMamba and VMamba—were evaluated and can be a variant of state-space neural networks designed for the efficient and stable modeling of long sequences.

All models were systematically evaluated on the NIH ChestX-ray14 dataset using the AUROC metric across 14 thoracic conditions, covering both common diseases like effusion and infiltration and less frequent ones such as hernia and fibrosis [8]. The results show that while Mamba-based models perform moderately on some classes, overall they lag behind top CNN and Transformer models like EfficientNet, CaFormer, and ConvFormer. These findings highlight the current strength and maturity of CNNs and Transformers for chest X-ray analysis and suggest that further improvements are needed before Mamba architectures can compete at the same level in clinical image classification tasks.

The remainder of this paper is organized as follows: Section 2 reviews the recent literature on deep learning applications in chest radiography. Section 3 details the materials and methods used, including the dataset, model architectures, and training protocols. Section 4 describes the evaluation metrics and experimental setup, followed by the presentation of model results. Section 5 provides an in-depth discussion and analysis of these results, including comparisons with prior studies. Finally, Section 6 summarizes the conclusions of the study and outlines the key contributions and implications for future research.

## 2. Related Works

Automated classification of thoracic diseases from chest radiographs is a well-established and rapidly evolving area in medical image analysis. With the introduction of large-scale labeled datasets, such as the NIH ChestX-ray14 dataset by Wang et al. [9], the application of deep learning techniques to chest X-ray (CXR) interpretation has become a prominent research focus. ChestX-ray14 contains over 112,000 frontal-view radiographs collected from more than 30,000 unique patients, annotated with 14 different thoracic disease labels using natural language processing (NLP) applied to radiology reports. The multi-label nature of the dataset, as well as the weak supervision in the label generation process, present unique challenges that have motivated a diverse set of methodological innovations.

Early deep learning approaches to chest X-ray classification primarily employed Convolutional Neural Networks (CNNs) for automated feature extraction and multi-label disease prediction. Wang et al. [9] established a baseline using a CNN architecture on the ChestX-ray14 dataset, demonstrating the potential of deep learning in detecting thoracic diseases under weak supervision. Building on this, Rajpurkar et al. introduced CheXNet [10], a 121-layer DenseNet pretrained on ImageNet, which achieved radiologist-level performance in pneumonia detection and inspired a wave of research focused on deeper architectures and transfer learning. For example, their work reported an AUC of 0.841 for overall classification when extended to 14 diseases. Subsequent studies explored more efficient backbones such as ResNet and Inception, with Tang et al. [11] demonstrating that CNN models can achieve high accuracy in differentiating normal from abnormal chest radiographs, reporting AUCs exceeding 0.98 on internal test sets.

Building upon these successes, more recent research has explored a variety of CNN architectures to balance improved accuracy with computational efficiency. For example, Ravi et al. (2022) proposed a multichannel EfficientNet ensemble combining models such as B0, B1, and B2, achieving high accuracy in lung disease detection across multiple chest X-ray datasets. Although their study did not specifically emphasize multi-label AUC scores on the NIH ChestX-ray14 dataset, it demonstrated the potential of systematic network scaling and ensemble methods to boost performance [12]. Meanwhile, lightweight architectures like MobileNet have attracted interest in their suitability in resource-constrained settings. Baltruschat et al. (2019) showed that a modified MobileNet V2 model could reach an average AUC of 0.811 across 14 thoracic pathologies in the NIH dataset, highlighting a practical trade-off between computational efficiency and diagnostic accuracy [13]. These developments underscore ongoing efforts to design flexible and effective CNN-based models tailored for diverse clinical environments.

In recent years, Transformer-based architectures have emerged as powerful alternatives to CNNs for medical image analysis, particularly due to their ability to model global contextual relationships in high-resolution data. Vision Transformers (ViTs), first introduced by Dosovitskiy et al. [14], have been successfully adapted to medical imaging tasks through fine-tuning on large-scale datasets. In the realm of chest X-ray classification, Taslimi et al. [7], developed SwinCheX, a model utilizing the Swin Transformer architecture. Their experiments on the NIH ChestX-ray14 dataset revealed an average AUC of 0.810 across 14 pathologies, surpassing several contemporary CNN-based methods. Similarly, Wollek et al. [15] explored the impact of training standard ViTs at higher resolutions, achieving a mean AUC of 0.842 and thus highlighting the importance of resolution in Transformer-based models for medical image classification. These studies underscore the growing interest and efficacy of Transformer-based models in advancing chest X-ray interpretation beyond the capabilities of conventional CNNs.

Recently, State Space Model (SSM)-based architectures such as Mamba have emerged as a compelling alternative to convolutional and Transformer-based backbones in visual tasks, offering a unique balance of efficiency and long-range context modeling [6]. These models, designed for linear-time sequence processing and selective information flow, are particularly well-suited to medical imaging, where high-resolution inputs and subtle anatomical patterns are common. In the context of chest X-ray analysis, Mamba’s ability to capture both local and global spatial dependencies is promising for interpreting diffuse pathologies across large visual fields. Vision-oriented variants such as MedMamba, proposed by Yue et al. [16], demonstrate this potential by combining grouped convolutions with bidirectional SSMs, achieving strong performance across a wide range of medical image datasets while maintaining low computational overhead—an essential trait for deployment in clinical environments. Similarly, Vision Mamba (Vim) [17] and VMamba [18] have shown that SSM-based backbones can outperform traditional CNNs and Transformers like DeiT and Swin in classification accuracy, efficiency, and memory usage, particularly at high input resolutions. While their application to chest X-ray datasets like NIH ChestX-ray14 remains emergent, the architectural features of Mamba variants—global receptive fields, linear complexity, and hardware efficiency—align well with the demands of chest radiograph interpretation, including multi-label disease classification and high-resolution abnormality detection.

The reviewed literature illustrates the evolution of deep learning models applied to chest radiography, particularly on the NIH ChestX-ray14 dataset. From early CNN-based models like CheXNet and DenseNet121 to recent Transformer and Mamba-based architectures, a broad range of approaches has been explored, each offering distinct advantages in feature extraction, scalability, or contextual modeling. CNNs have remained robust and efficient for thoracic anomaly detection, while Transformer-based models have improved the global contextual understanding and multi-label discrimination. The emergence of Mamba-based architectures brings promising innovations in linear-time sequence modeling and hardware efficiency, though their application to high-resolution medical imaging remains relatively new and underexplored.

Despite the individual successes of these models, existing studies often evaluate them in isolation or under varying conditions, limiting direct comparability. To address this gap, a unified and fair benchmarking of 14 models across the CNN, Transformer, and Mamba architecture families is presented. Each was trained and evaluated under identical experimental protocols. By doing so, the first comprehensive performance landscape that directly compares these architectures is provided on the same clinical imaging benchmark, highlighting their relative strengths, limitations, and suitability for deployment in real-world AI-assisted diagnostic pipelines.

## 3. Materials and Methods

### 3.1. Dataset Description

This study primarily utilizes the NIH ChestX-ray14 dataset (NIH Clinical Center, ABD), a widely recognized public resource that has significantly advanced deep learning for thoracic disease detection [8]. It contains 112,120 frontal chest X-ray images from 30,805 unique patients, with each image provided at a resolution of 1024 × 1024 pixels. The dataset is annotated with fourteen common thoracic conditions, including atelectasis, cardiomegaly, effusion, pneumonia, and more. These labels were automatically generated using natural language processing (NLP) techniques applied to radiology reports. A key feature of this dataset is its multi-label nature, meaning that a single image can be associated with multiple diseases. This requires models not only to identify individual pathologies but also to learn the relationships and co-occurrence patterns among different conditions.

Despite its scale and importance, the NIH ChestX-ray14 dataset has some limitations that influence model development and evaluation. The NLP-derived labels, while generally reliable, have an estimated accuracy of around 90%, introducing some label noise. Moreover, detailed bounding box annotations for precise disease localization are scarce, limiting supervised training for detection tasks. There is also a significant class imbalance, as certain diseases appear much more frequently than others, posing challenges for model training.

**Figure 2:**
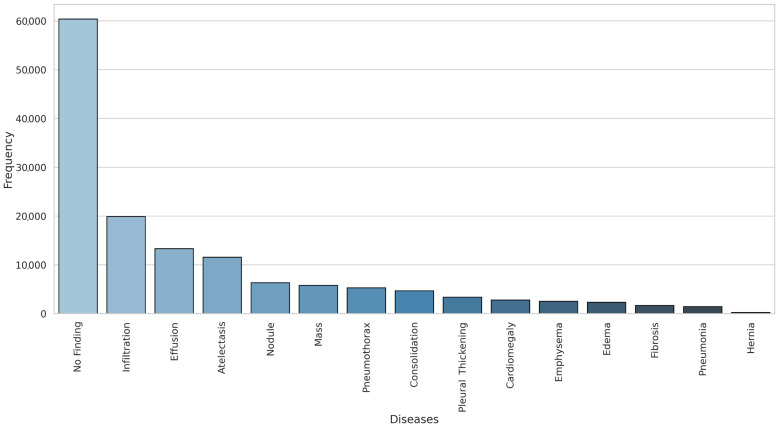
Disease frequency distribution in the ChestX-ray14 dataset.

For training, mild data augmentation and preprocessing were applied to enhance model robustness without introducing drastic distortions. Images were resized to 224 × 224 pixels for a consistent input size, followed by a random horizontal flip applied with a 50% probability to simulate natural variations in image orientation. After these steps, images were converted to tensors and normalized using dataset-specific mean and standard deviation values. To evaluate the model, the dataset was randomly split into training (70%), validation (10%), and test (20%) sets, ensuring that patients did not overlap across splits, following the protocol established in previous studies to prevent data leakage and ensure a fair performance assessment. Lastly, the dataset’s frequency distribution across the fourteen disease categories is illustrated in Figure 2 below using a bar chart.

### 3.2. Model Architectures

In this study, a comprehensive comparative evaluation of fourteen state-of-the-art deep learning models is performed spanning three main architectural families: Convolutional Neural Networks (CNNs), Transformer-based models, and State Space Models (Mamba variants). These architectures were carefully chosen due to their strong performance documented in the recent literature, diverse inductive biases, and varying computational complexities, reflecting different strengths and trade-offs relevant to clinical deployment. By examining these varied approaches, architectures best suited for robust and efficient thoracic disease classification in chest X-rays are aimed to be identified, considering both accuracy and practical constraints such as inference speed and resource usage.

To ensure a fair and unbiased comparison, all models were trained and tested on the NIH ChestX-ray14 dataset using consistent preprocessing steps, standardized data splits, and identical training configurations. This uniform setup minimizes confounding factors, allowing performance differences to be attributed primarily to architectural design choices. In the following sections, the key characteristics and underlying motivations for each model family are described, providing insights into how their unique mechanisms address the challenges of multi-label chest X-ray classification.

#### 3.2.1. Convolutional Neural Networks (CNNs)

Convolutional Neural Networks (CNNs) have been foundational in medical image analysis, especially for chest radiography, due to their proven ability to capture local spatial hierarchies and textures crucial for identifying thoracic abnormalities. Their relatively efficient computation and well-understood architecture make them a natural starting point for deep learning in medical imaging. This study evaluates six prominent CNN architectures, selected for their distinct design innovations and demonstrated efficacy in multi-label disease classification:

- DenseNet121 (8 M parameters)—DenseNet’s dense connectivity pattern, where each layer receives inputs from all preceding layers, promotes feature reuse and mitigates the vanishing gradient problem. This dense information flow enables the model to learn richer and more diverse features with fewer parameters [19]. DenseNet121′s success in CheXNet, which achieved radiologist-level pneumonia detection on NIH ChestX-ray14, illustrates its capability to capture subtle radiographic patterns critical for thoracic disease identification [10].
- ResNet34 (21 M)—ResNet’s introduction of residual connections fundamentally improved the trainability of deep networks by allowing gradients to bypass several layers, addressing degradation problems in very deep architectures. ResNet34 strikes a balance between depth and complexity, offering stable training and generalization. Its residual blocks help the model learn both low-level textures and higher-order semantic features, which are essential for differentiating overlapping pathologies in chest X-rays [20].
- InceptionV3 (24 M)—Inception architectures utilize parallel convolutional filters of varying sizes (e.g., 1 × 1, 3 × 3, and 5 × 5) within inception modules to capture multi-scale spatial features simultaneously. This design enables the network to efficiently aggregate local and global contextual information, enhancing its ability to discern complex lung patterns such as nodules, masses, or infiltrates. InceptionV3 has shown robustness in handling the heterogeneous data distributions commonly found in medical images [21].
- ResNext50 (25 M)—ResNext employs grouped convolutions to increase cardinality—the number of independent paths through the network—thereby boosting representational power without a proportional increase in computational cost. This architecture excels in multi-label tasks by allowing the model to capture diverse disease features in parallel. ResNext50’s modular design facilitates flexible scaling and has demonstrated strong performance in thoracic abnormality classification benchmarks [22].
- EfficientNet-B0 (5.3 M)—EfficientNet introduces a compound scaling method that uniformly scales the network depth, width, and input resolution based on a fixed set of scaling coefficients. This approach yields models that achieve high accuracy with fewer parameters and less computational overhead. EfficientNet-B0’s efficiency and scalability are particularly advantageous in medical imaging contexts, where high-resolution images and resource constraints pose challenges [23].
- MobileNetV4 (6 M)—MobileNet architectures are tailored for lightweight deployment using depthwise separable convolutions to drastically reduce the model size and computational cost. MobileNetV4 further integrates novel architectural improvements such as squeeze-and-excitation modules and optimized activation functions, maintaining competitive accuracy with low latency. This makes MobileNetV4 suitable for clinical environments requiring fast inference on limited hardware, such as bedside diagnostics or portable X-ray devices [24].

#### 3.2.2. Transformer-Based Models

Originally developed for natural language processing, Transformers have recently made significant inroads into computer vision through architectures like the Vision Transformer (ViT) and its numerous derivatives. Their core innovation—self-attention—enables global receptive fields, allowing models to capture long-range dependencies across the entire image. This feature is particularly valuable for chest X-ray interpretation, where diseases often present with diffuse, overlapping, or spatially dispersed patterns that are difficult to model with traditional convolutional filters. In this study, several representative Transformer-based models that reflect the diversity and progression of this architectural family are evaluated:

- DaViT-Tiny (28 M)—The Dual Attention Vision Transformer introduces a novel combination of spatial and channel-wise attention mechanisms. This dual-path attention structure improves the model’s ability to capture both global structure and fine-grained details, making it especially effective for multi-label classification where lesions may differ significantly in size and location [25].
- ConvFormer (28 M)—A hybrid architecture, ConvFormer blends the local feature extraction strengths of CNNs with the long-range modeling capabilities of Transformers. By integrating convolutional layers within Transformer blocks, it maintains strong spatial inductive biases while also capturing contextual dependencies—a beneficial combination for medical images that contain both localized and global pathological features [26].
- CaFormer (~29 M)—The Conditional Attention Transformer (CaFormer) is a model introduced within the MetaFormer framework, which abstracts the architecture of Transformers to focus on the token mixer component. CaFormer enhances its adaptability by employing depthwise separable convolutions as token mixers in the initial stages and transitioning to vanilla self-attention in the later stages. This hybrid approach allows the model to effectively capture both local and global dependencies [26].
- DeiT (22 M)—The Data-Efficient Image Transformer was designed to make training ViTs feasible on smaller datasets through knowledge distillation. This is especially relevant in the medical domain, where large-scale, high-quality annotated data can be scarce. DeiT maintains competitive performance while being more computationally accessible than traditional ViT [27].
- Swin Transformer v1/v2 (29–60 M)—Swin Transformers adopt a hierarchical architecture with shifted window-based self-attention. This design preserves the Transformer’s global modeling capacity while dramatically improving efficiency and scalability for high-resolution images. The model builds representations at multiple scales, closely mimicking the way radiologists zoom in and out across regions of interest in chest X-rays [28,29].

#### 3.2.3. Mamba-Based State Space Models

Mamba-based architectures represent an emerging class of sequence modeling frameworks grounded in Structured State Space Models (S4). Unlike traditional convolutional or attention-based architectures, Mamba models are designed to operate in linear time with respect to the input length, offering an appealing balance between expressiveness, memory efficiency, and hardware scalability. These characteristics make them particularly well-suited for high-resolution medical imaging tasks, where long-range dependencies and efficient processing are critical.

- VMamba (~22M)—VMamba extends the Mamba language model to the vision domain by introducing a Selective-Scan 2D (SS2D) module. This component enables the model to perform directional scanning in multiple spatial paths, allowing global receptive field modeling with linear complexity. VMamba has demonstrated strong accuracy, throughput, and memory efficiency in standard computer vision benchmarks. However, its classification performance on medical images has shown variability, especially when compared to mature Transformer-based counterparts [18].
- MedMamba (~14M)—MedMamba is the first Mamba variant specifically adapted for generalized medical image classification. It employs a hybrid design combining grouped convolutions with SSM-based processing, as well as medical domain-specific normalization techniques to better align with clinical imaging characteristics. Its lightweight design and theoretical scalability make it attractive for real-time or resource-constrained settings. Despite these advantages, it is indicated by the evaluations that MedMamba underperforms relative to CNN and Transformer baselines on complex thoracic anomalies, highlighting ongoing challenges in adapting Mamba architectures to the unique demands of medical image analysis [10].

Beyond the general architectural description, adapting Mamba variants to medical imaging introduces several unique considerations. Chest radiographs frequently exhibit subtle grayscale variations, overlapping anatomical structures, and high-resolution details, which require models capable of capturing localized textures and long-range contextual dependencies. VMamba addresses this need through the Selective-Scan 2D (SS2D) mechanism, which enables linear-time modeling of extended spatial dependencies across the thoracic region. This feature is particularly relevant for diffuse abnormalities, such as emphysema or interstitial fibrosis, that span large anatomical areas.

MedMamba builds upon these principles by integrating grouped convolutions and domain-specific normalization layers designed explicitly for medical images. These modifications aim to enhance robustness against noise, intensity variations, and contrast inconsistencies commonly encountered in clinical radiographs. Furthermore, its lightweight design and reduced computational footprint highlight its potential suitability for real-time deployment in resource-limited environments, such as portable radiography systems or low-infrastructure healthcare settings.

Despite these advantages, several implementation challenges remain. The weakly labeled nature of the NIH ChestX-ray14 dataset limits the ability of Mamba models to fully exploit their long-range modeling capabilities, as noisy or imprecise labels may disrupt sequential dependency learning. In addition, training stability and hyperparameter sensitivity are more pronounced than in established CNN and Transformer counterparts, which currently restricts their competitiveness. These findings suggest that while Mamba-based models hold strong theoretical promise for medical image analysis, further architectural refinements and access to curated, high-quality datasets are necessary to unlock their full potential.

Table 2 provides a consolidated overview of the fourteen deep learning architectures benchmarked in this study, grouped by model category. The table highlights the diverse architectural strategies evaluated—ranging from traditional convolutional networks to modern attention-based Transformers and emerging state space models like Mamba. CNN-based models such as DenseNet121, ResNet34, and EfficientNet-B0 offer well-established baselines with relatively small parameter counts and computational efficiency. Transformer-based models, including ConvFormer and CaFormer, introduce global context modeling and scalable attention mechanisms that contribute to superior performance on complex spatial patterns found in chest radiographs. Mamba-based models, although newer and less explored, are represented by VMamba and MedMamba, which incorporate linear-time state space modeling tailored for visual sequences. This table underscores the diversity of design philosophies across architectures, providing essential context for interpreting their comparative performance results in later sections of this paper.

This diverse selection of models captures the evolution and innovation within deep learning for vision tasks—ranging from the well-established convolutional neural networks (CNNs), through the recent surge of Transformer-based architectures, to the emerging State Space Models like Mamba variants. By including these fundamentally different design paradigms, a comprehensive and robust empirical comparison is offered that highlights how distinct architectural choices impact model capabilities. Evaluating all models under consistent training conditions and standardized datasets ensures a fair and unbiased assessment of their strengths and limitations. This approach not only sheds light on the intricate relationship between model structure and diagnostic accuracy in complex multi-label chest X-ray classification but also provides practical guidance for both researchers and clinicians, ultimately supporting informed decisions in AI model development and clinical deployment.

## 4. Results

The empirical evaluation conducted in this study involved a thorough comparative analysis of state-of-the-art deep learning architectures’ performance on the NIH ChestX-ray14 dataset. The primary metric used for the performance assessment was the Area Under the Receiver Operating Characteristic Curve (AUROC), which measures a model’s ability to distinguish between positive and negative cases across a range of classification thresholds. In the context of medical image analysis, particularly for imbalanced datasets like ChestX-ray14, AUROC is a well-established indicator of model effectiveness, with values above 0.80 typically considered clinically meaningful.

This study benchmarked three primary categories of models: Convolutional Neural Networks (CNNs), Transformer-based architectures, and Mamba-based state space models. The CNN group included DenseNet121, ResNet34, InceptionV3, ResNext50, EfficientNet, and MobileNetV4. The Transformer-based models evaluated included DaViT-Tiny, ConvFormer, CaFormer, DeiT, and Swin Transformer (v1 and v2). The Mamba-based models tested were VMamba and MedMamba. Performance results for all models are summarized in Table 3.

Contrary to earlier hypotheses suggesting that Mamba-based models might provide a performance advantage due to their efficient handling of sequential information and long-range dependencies, the empirical results revealed that these models were generally outperformed by both CNN and Transformer counterparts. VMamba and MedMamba underperformed in most anomaly categories, particularly in spatially complex or rare pathologies such as nodules (AUROC: 0.63 and 0.65, respectively) and infiltration (0.68 for both). While MedMamba achieved moderate performance in cardiomegaly (0.89) and edema (0.87), these results still fell short of the top-performing Transformer-based models like ConvFormer and CaFormer, which consistently scored higher or matched the best values across most conditions.

The best performances were achieved by Transformer-based architectures. Notably, CaFormer obtained the highest AUROC for atelectasis (0.83), and ConvFormer achieved the highest or tied scores for mass (0.85), effusion (0.88), and pleural thickening (0.78). EfficientNet, a CNN model, also demonstrated exceptional reliability, especially for hernia (0.94) and edema (0.89). Across the full model landscape, ConvFormer, CaFormer, and EfficientNet emerged as the most robust and consistent models for multi-label chest X-ray anomaly detection.

In contrast to earlier expectations, the results suggest that the current implementations of Mamba-based architectures are not yet optimized for high-resolution visual pattern recognition tasks such as chest X-ray classification. Although Mamba architectures theoretically offer computational advantages like linear-time complexity, this benefit did not translate into performance superiority in practice under the current training and evaluation settings.

These findings highlight the critical importance of architecture choice and suggest that Transformer-based models—particularly ConvFormer and CaFormer—represent the most reliable solutions for clinical-grade automated CXR interpretation.

To further emphasize the role of the class imbalance, performance outcomes were analyzed by grouping thoracic diseases into common versus rare categories. Common pathologies, including effusion, infiltration, and atelectasis, exhibited relatively stable AUROC values across most architectures, with Transformers such as ConvFormer and CaFormer consistently achieving near or above 0.80. In contrast, rare classes such as hernia, fibrosis, and pneumonia revealed greater variability in performance, reflecting the challenges posed by limited training samples. EfficientNet achieved the highest AUROC of 0.94 for hernia, despite its extremely low prevalence (0.2%), demonstrating that specific architectures can generalize well even under severe class imbalance. These results indicate that model selection should consider the overall mean AUROC and performance across rare yet clinically significant pathologies, as these categories may disproportionately impact diagnostic utility in real-world practice.

To assess whether the observed performance differences were statistically significant, 95% confidence intervals (CIs) for AUROC values were calculated using bootstrap resampling (1000 iterations) across all 14 classes. Additionally, the DeLong test was applied for pairwise AUROC comparisons between top-performing models (ConvFormer, CaFormer, and EfficientNet-B0) and Mamba-based models (VMamba and MedMamba). The results indicate that ConvFormer and CaFormer consistently outperformed VMamba and MedMamba, with statistically significant differences (*p* < 0.01). Comparisons between ConvFormer and EfficientNet-B0 showed smaller but significant differences in certain classes such as mass and effusion (*p* < 0.05), while for high-performing classes such as hernia and emphysema, performance differences were not statistically significant (*p* > 0.05). These findings confirm that the superior performance of hybrid Transformer–CNN architectures is robust and not due to random variation.

In addition to diagnostic accuracy, training and inference efficiency are crucial for clinical deployment. Table 4 summarizes the computational profiles of the evaluated architectures, including parameter counts, floating-point operations per second (FLOPs), average training time per epoch, and inference latency measured on an NVIDIA A100 GPU.

The results confirm that while ConvFormer and CaFormer deliver the strongest accuracy, their computational footprint is higher compared to CNNs. Mamba-based models demonstrate favorable efficiency with substantially lower FLOPs and latency, supporting their potential use in real-time or resource-constrained clinical environments, despite their current accuracy limitations.

**Figure 3:**
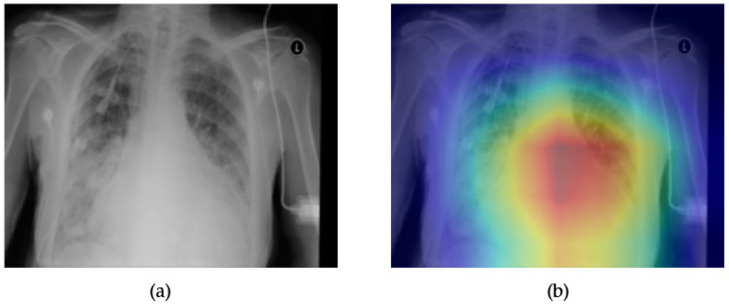
Comparison between the original chest X-ray and its Grad-CAM heatmap highlighting the detected cardiomegaly region. (**a**) Original chest X-ray; (**b**) Grad-CAM heatmap.

**Figure 4:**
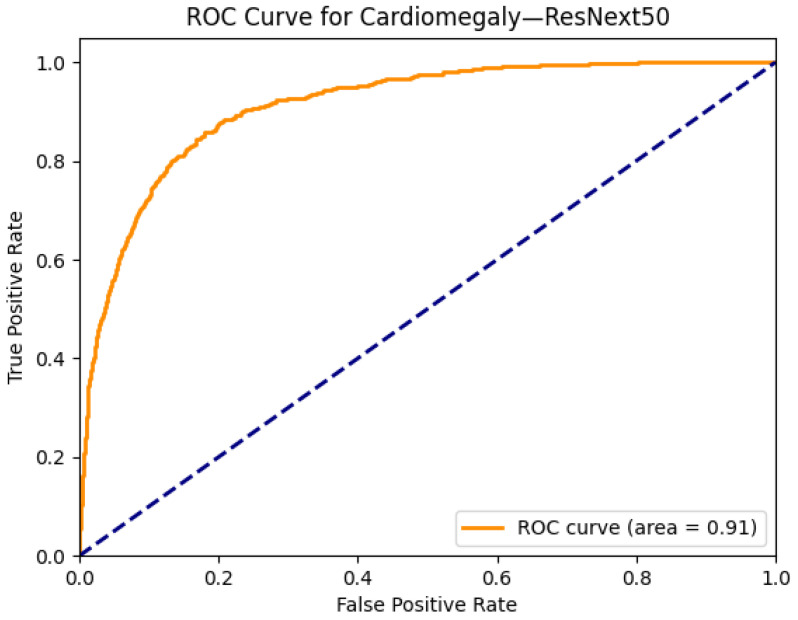
ROC curve for cardiomegaly using the ResNext50 model. The area under the curve (AUC) is 0.91, indicating strong classification performance.

Figure 3 and Figure 4 illustrate both the model interpretability and discriminative performance for cardiomegaly detection using the ResNext50 architecture. In Figure 3a, the original chest X-ray is presented alongside its corresponding Grad-CAM heatmap in Figure 3b, which highlights the model’s regions of focus during prediction. The activated area in the Grad-CAM visualization aligns well with the anatomical location of the heart, suggesting that the model is learning meaningful and clinically relevant features to identify cardiomegaly. This enhances the interpretability of the model and provides an additional layer of trust for clinical decision support.

Figure 4 presents the ROC curve for cardiomegaly classification by ResNext50, achieving an area under the curve (AUC) of 0.91, which indicates excellent discriminative performance. The curve demonstrates high sensitivity and specificity across thresholds, confirming the model’s robustness for this particular condition. The combined use of explainability (via Grad-CAM) and quantitative evaluation (via AUC) highlights ResNext50’s ability to not only perform well but also offer interpretable and clinically plausible outputs.

**Figure 5:**
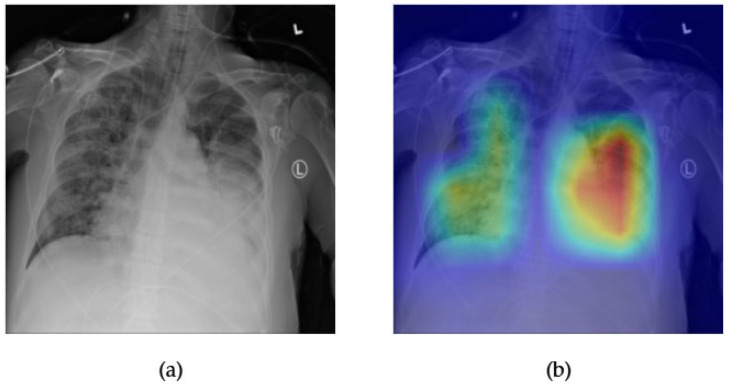
Comparison between the original chest X-ray and its Grad-CAM heatmap highlighting the detected consolidation region. (**a**) Original chest X-ray; (**b**) Grad-CAM heatmap.

**Figure 6:**
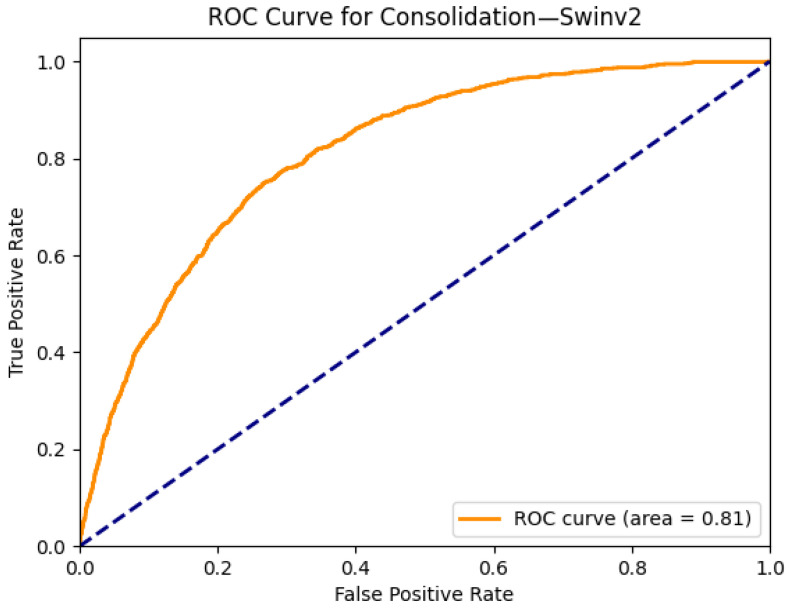
ROC curve for consolidation using the Swinv2 model. The area under the curve (AUC) is 0.81, indicating strong classification performance.

Figure 5 and Figure 6 illustrate the explainability and classification performance of the SwinV2 model for the detection of consolidation in chest radiographs. In Figure 5a, the frontal chest X-ray shows a subtle increase in opacity in the right lower and left mid-lung zones, which are indicative of potential consolidation. The corresponding Grad-CAM heatmap Figure 5b highlights these areas of increased density, suggesting that the model accurately attends to the relevant regions associated with this pathology. The alignment between radiological signs and model attention maps enhances the interpretability and clinical reliability of the prediction.

Figure 6 displays the ROC curve for SwinV2 in predicting consolidation. The model achieves an AUC of 0.81, indicating good discriminative ability. While not as high as some other pathologies, this level of performance is consistent with the inherent diagnostic complexity of detecting consolidation in chest X-rays, especially when it overlaps with other findings or presents with diffuse patterns. The results demonstrate that SwinV2 provides a robust balance between classification accuracy and localization interpretability for this challenging class.

**Figure 7:**
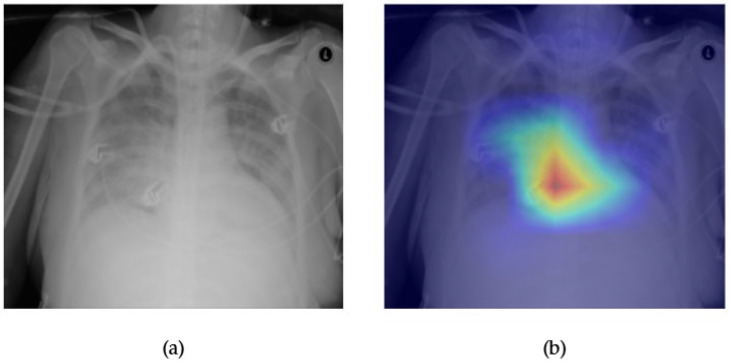
Comparison between the original chest X-ray and its Grad-CAM heatmap highlighting the detected edema region. (**a**) Original chest X-ray; (**b**) Grad-CAM heatmap.

**Figure 8:**
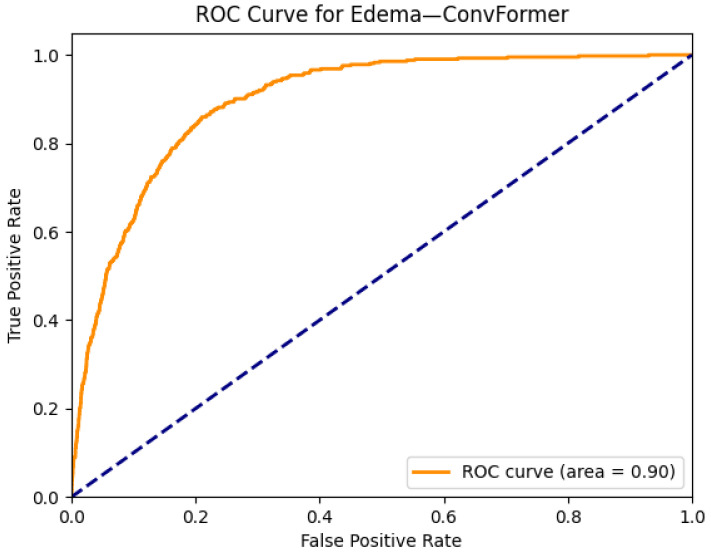
ROC curve for edema using the ConvFormer model. The area under the curve (AUC) is 0.90, indicating strong classification performance.

Figure 7 and Figure 8 demonstrate the explainability and classification performance of the ConvFormer model in detecting pulmonary edema in chest radiographs. In Figure 7a, the frontal chest X-ray presents bilateral haziness in the lower lung fields, a common radiographic manifestation of edema. The corresponding Grad-CAM heatmap in Figure 7b accurately highlights the central and lower lung zones, which is consistent with typical fluid accumulation in cases of pulmonary congestion. This spatial correlation suggests that ConvFormer effectively localizes diagnostically relevant regions, supporting its clinical interpretability.

Figure 8 illustrates the ROC curve for edema classification using the ConvFormer model, with an AUC of 0.90, indicating strong discriminative performance. This high score reflects the model’s capacity to differentiate edema cases from non-edema instances across a range of decision thresholds. When considered together, the localization fidelity provided by Grad-CAM and the high AUROC validate ConvFormer’s utility in not only classifying but also justifying its predictions in a clinically meaningful manner.

**Figure 9:**
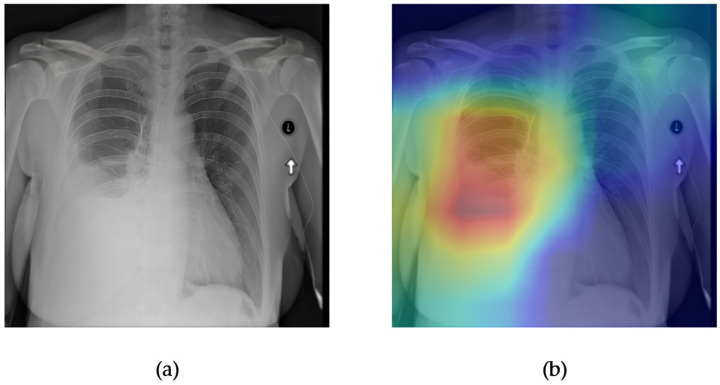
Comparison between the original chest X-ray and its Grad-CAM heatmap highlighting the detected effusion region. (**a**) Original chest X-ray; (**b**) Grad-CAM heatmap.

**Figure 10:**
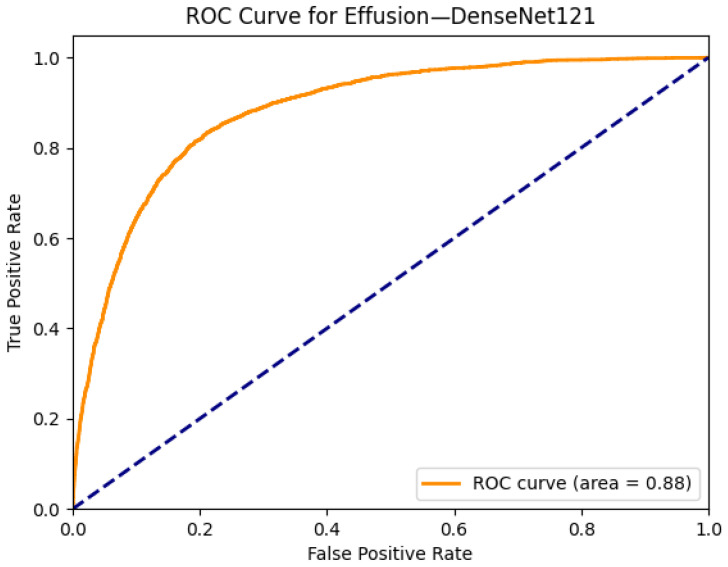
ROC curve for effusion using the DenseNet121 model. The area under the curve (AUC) is 0.88, indicating strong classification performance.

Figure 9 and Figure 10 illustrate both the interpretability and predictive performance of the DenseNet121 model for pleural effusion detection. The frontal chest X-ray shown in Figure 9a displays subtle blunting of the costophrenic angle and increased opacity over the left lower lung zone, which are consistent with pleural fluid accumulation. The accompanying Grad-CAM heatmap in Figure 9b highlights this region with high intensity, demonstrating that DenseNet121 correctly attends to clinically relevant areas associated with effusion. This alignment reinforces the reliability of the model’s internal feature attribution.

Figure 10 presents the ROC curve for effusion classification by DenseNet121, yielding an AUC of 0.88, which indicates strong discriminative performance. While slightly lower than some Transformer-based models evaluated in this study, DenseNet121 offers a favorable balance between accuracy, interpretability, and computational efficiency. The combined visualization and performance metrics confirm that this CNN architecture remains a competitive and trustworthy choice for multi-label thoracic disease detection in clinical-grade datasets such as ChestX-ray14.

**Figure 11:**
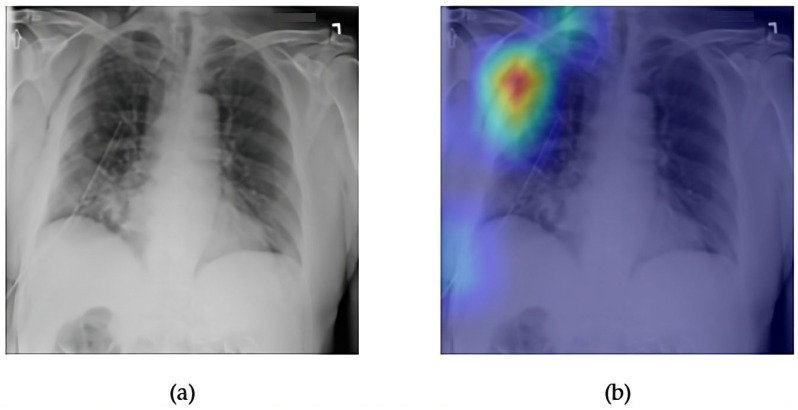
Comparison between the original chest X-ray and its Grad-CAM heatmap highlighting the detected emphysema region. (**a**) Original chest X-ray; (**b**) Grad-CAM heatmap.

**Figure 12:**
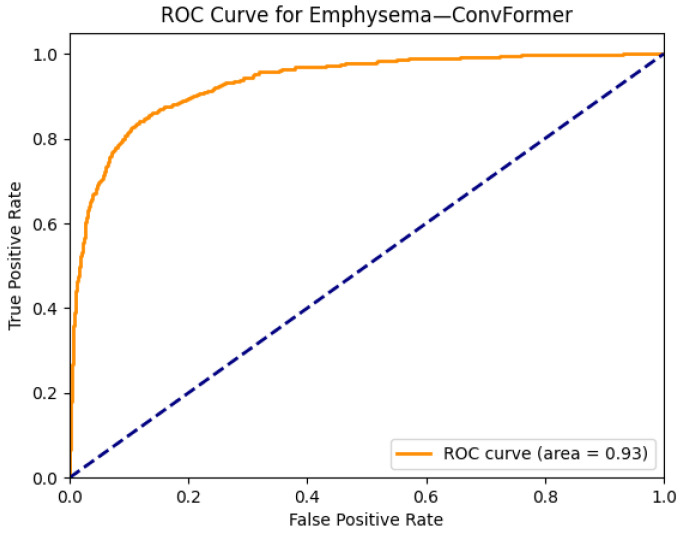
ROC curve for emphysema using the ConvFormer model. The area under the curve (AUC) is 0.93, indicating strong classification performance.

Figure 11 and Figure 12 present the explainability and classification performance of the ConvFormer model in detecting emphysema, a pathology characterized by hyperinflation and reduced vascular markings in the lung fields. The frontal chest radiograph in Figure 11a displays increased radiolucency, particularly in the upper lung zones, which are common signs of emphysematous changes. The corresponding Grad-CAM heatmap (Figure 11b strongly activates the upper lobe regions, indicating that the model accurately focuses on the relevant anatomical zones known to exhibit early emphysema manifestations.

In Figure 12, the ROC curve for ConvFormer on the emphysema class reveals an AUC of 0.93, reflecting excellent discriminative capability. This high performance underscores ConvFormer’s ability to generalize well, even for diseases that often present with subtle or diffuse radiographic patterns. The strong alignment between Grad-CAM activation and known emphysema zones, combined with the high AUROC, demonstrate ConvFormer’s effectiveness in both classification accuracy and interpretable decision-making for this clinically significant condition.

**Figure 13:**
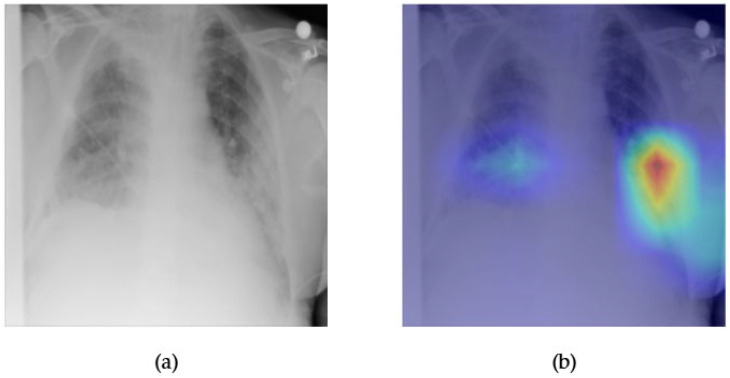
Comparison between the original chest X-ray and its Grad-CAM heatmap highlighting the detected infiltration region. (**a**) Original chest X-ray; (**b**) Grad-CAM heatmap.

**Figure 14:**
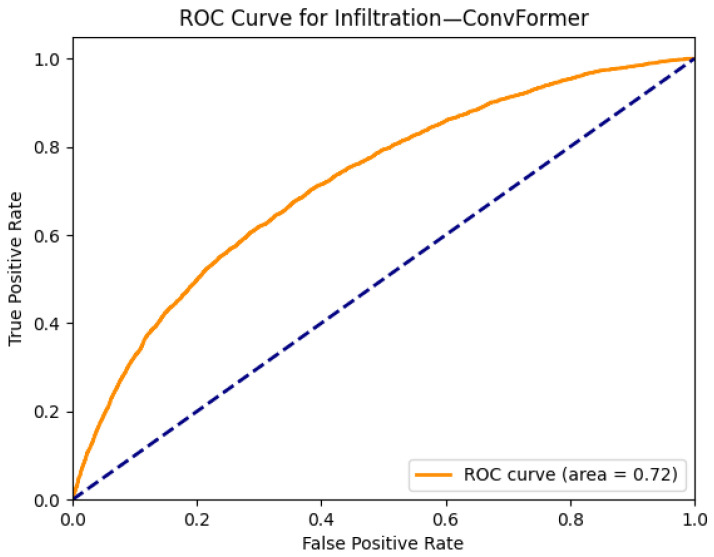
ROC curve for infiltration using the ConvFormer model. The area under the curve (AUC) is 0.72, indicating strong classification performance.

Figure 13 and Figure 14 illustrate the visual explanation and performance of the ConvFormer model for detecting pulmonary infiltration, a non-specific radiological finding that can represent infection, inflammation, or fluid accumulation. The original chest X-ray in Figure 13a shows subtle patchy opacities in the right mid-to-lower lung zone, which may correspond to infiltration. The Grad-CAM heatmap in Figure 13b reveals focused activation around this region, suggesting that ConvFormer attends to areas relevant for this pathology. While Grad-CAM provides interpretable evidence of the model’s localization capability, some diffuse patterns typical of infiltration may challenge precise delineation.

Figure 14 shows the ROC curve for ConvFormer on the infiltration class, yielding an AUC of 0.72. Compared to other conditions evaluated in this study, this is relatively lower and reflects the diagnostic complexity of infiltration, where radiological features can overlap with multiple pathologies and exhibit variability in presentation. Nevertheless, the model demonstrates reasonable sensitivity and a useful level of discrimination. The moderate performance combined with interpretable Grad-CAM visualization highlights the need for continued refinement in handling ambiguous or overlapping thoracic findings.

**Figure 15:**
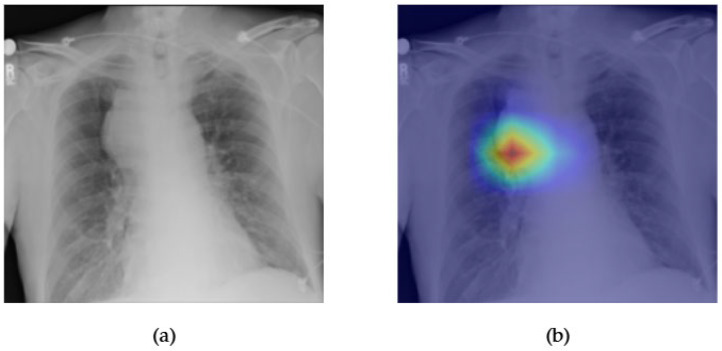
Comparison between the original chest X-ray and its Grad-CAM heatmap highlighting the detected mass region. (**a**) Original chest X-ray; (**b**) Grad-CAM heatmap.

**Figure 16:**
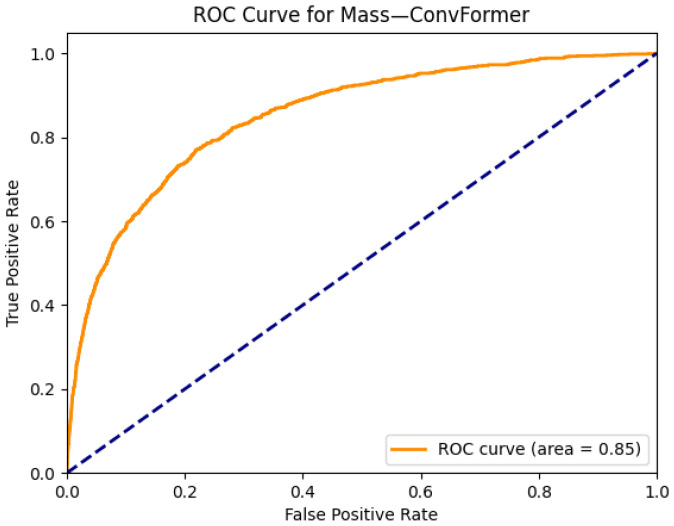
ROC curve for a mass using the ConvFormer model. The area under the curve (AUC) is 0.85, indicating strong classification performance.

Figure 15 and Figure 16 present the interpretability and classification performance of the ConvFormer model in detecting pulmonary masses in chest radiographs. In Figure 15a, the original X-ray reveals a well-defined opacity in the right mid-lung zone, consistent with the radiographic appearance of a solitary pulmonary mass. The Grad-CAM heatmap in Figure 15b shows strong activation precisely over this region, indicating that ConvFormer successfully localizes the relevant area of concern. This interpretability is crucial in clinical applications, where explainable AI outputs can support radiologist validation and trust.

In Figure 16, the ROC curve for mass detection using ConvFormer yields an AUC of 0.85, reflecting strong discriminative power. Although slightly lower than the AUCs observed for more prevalent conditions like edema or emphysema, this result remains clinically significant given the complexity and heterogeneity of masses in chest imaging. Together, the heatmap and classification curve confirm that ConvFormer not only identifies mass lesions with high accuracy but also focuses its attention on radiologically meaningful regions, making it a promising tool for assisting radiologists in the early detection of potentially malignant thoracic abnormalities.

**Figure 17:**
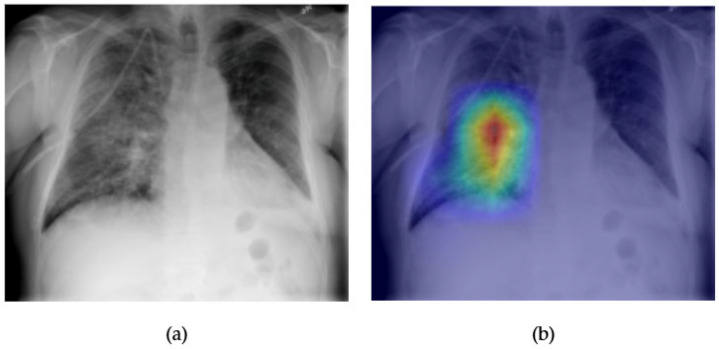
Comparison between the original chest X-ray and its Grad-CAM heatmap highlighting the detected nodule region. (**a**) Original chest X-ray; (**b**) Grad-CAM heatmap.

**Figure 18:**
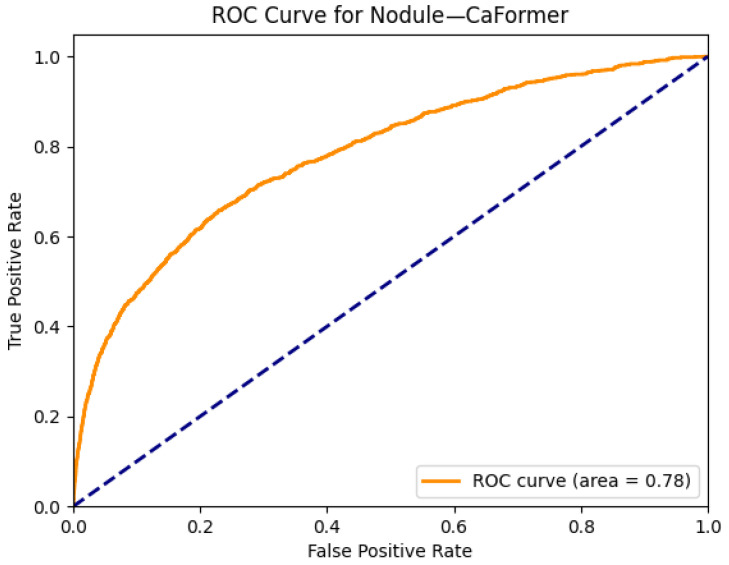
ROC curve for a nodule using the CaFormer model. The area under the curve (AUC) is 0.78, indicating strong classification performance.

Figure 17 and Figure 18 illustrate the localization and classification performance of the CaFormer model for detecting pulmonary nodules, which are often small, subtle, and radiologically challenging to detect. In the original chest X-ray shown in Figure 17a, a small opacity is seen in the right mid-lung zone. The Grad-CAM visualization in Figure 17b highlights this same region with strong activation, indicating that CaFormer successfully identifies and focuses on the radiographic area most consistent with the presence of a nodule. This spatial alignment enhances the interpretability of the model and demonstrates its ability to attend to clinically relevant features.

The ROC curve presented in Figure 18 shows an AUC of 0.78 for the nodule class. While this is lower than AUCs observed for larger or more radiographically distinct pathologies such as edema or emphysema, it still represents a strong classification performance for a class that is known to present subtle radiological findings. These results underscore CaFormer’s capacity to generalize across both common and diagnostically challenging classes, offering a balance between accuracy and explainability in multi-label thoracic disease detection.

**Figure 19:**
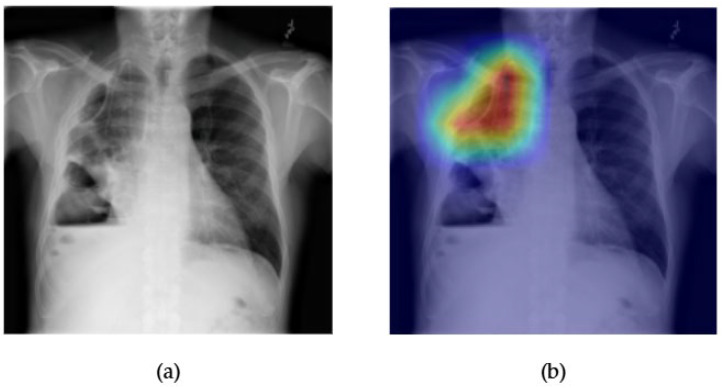
Comparison between the original chest X-ray and its Grad-CAM heatmap highlighting the detected region with pleural thickening. (**a**) Original chest X-ray; (**b**) Grad-CAM heatmap.

**Figure 20:**
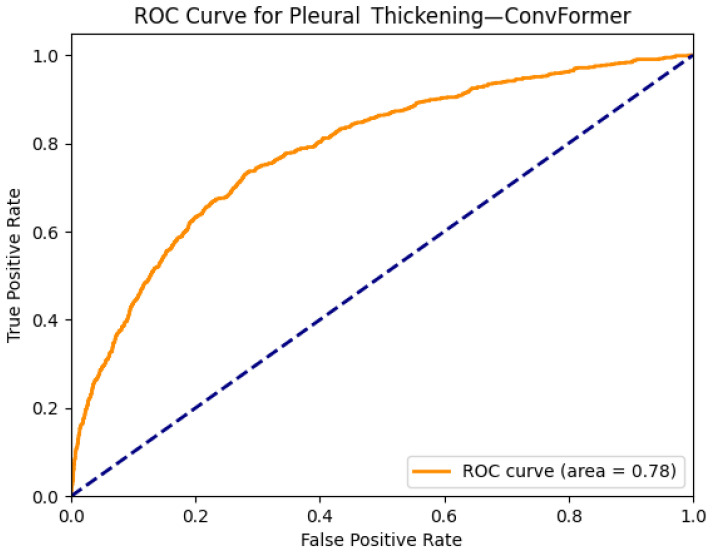
ROC curve for pleural thickening using the ConvFormer model. The area under the curve (AUC) is 0.78, indicating strong classification performance.

Figure 19 and Figure 20 showcase the interpretability and performance of the ConvFormer model in detecting pleural thickening, a subtle thoracic abnormality often seen along the lateral or apical pleural margins. In Figure 19a, the original chest X-ray displays an area of linear density along the left upper hemithorax, which could be suggestive of a pleural-based pathology. The corresponding Grad-CAM heatmap Figure 19b reveals prominent activation in this region, indicating that ConvFormer effectively localizes features relevant to pleural thickening. The heatmap’s spatial focus provides reassurance that the model bases its predictions on clinically meaningful anatomical structures.

The ROC curve in Figure 20 presents an AUC of 0.78 for the pleural thickening class. Although not as high as results for more visually distinct pathologies like edema or emphysema, this performance remains notable given the subtle and variable radiographic presentation of pleural thickening. These findings suggest that ConvFormer maintains a solid balance between predictive capability and explainability, even for harder-to-detect conditions, underscoring its generalizability across diverse thoracic anomalies

**Figure 21:**
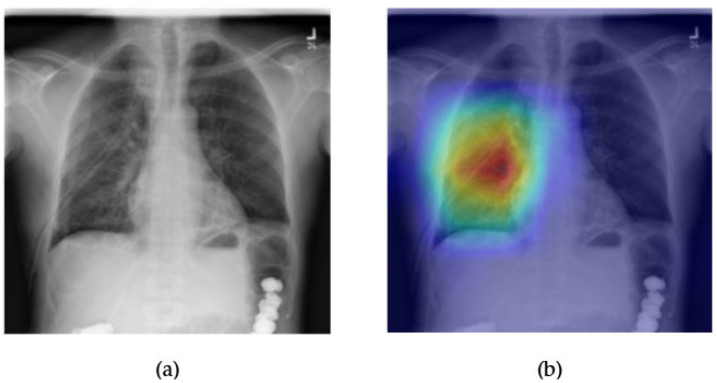
Comparison between the original chest X-ray and its Grad-CAM heatmap highlighting the detected pneumothorax region. (**a**) Original chest X-ray; (**b**) Grad-CAM heatmap.

**Figure 22:**
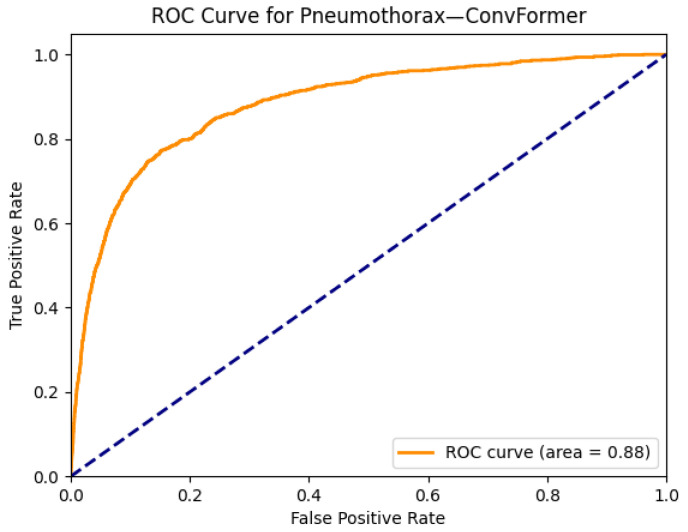
ROC curve for pneumothorax using the ConvFormer model. The area under the curve (AUC) is 0.88, indicating strong classification performance.

Figure 21 and Figure 22 demonstrate the explainability and performance of the ConvFormer model in detecting pneumothorax, a critical condition that presents as a collapsed lung and appears as an area of increased lucency with an absence of lung markings. In Figure 21a, the chest X-ray shows subtle radiolucency in the right hemithorax, suggestive of a pneumothorax. The Grad-CAM heatmap in Figure 21b highlights this region with strong activation, indicating that ConvFormer successfully attends to relevant radiographic features. The model’s ability to localize the pneumothorax zone is especially valuable in clinical settings where timely identification is crucial.

The ROC curve in Figure 22 shows an AUC of 0.88, reflecting robust classification performance. This high level of accuracy supports ConvFormer’s effectiveness in identifying pneumothorax, which can be particularly difficult when the presentation is subtle or confounded by artifacts such as medical devices or patient positioning. The combination of strong predictive metrics and localized visual focus demonstrates ConvFormer’s potential as a clinically reliable tool for detecting emergent thoracic conditions in chest radiography.

**Figure 23:**
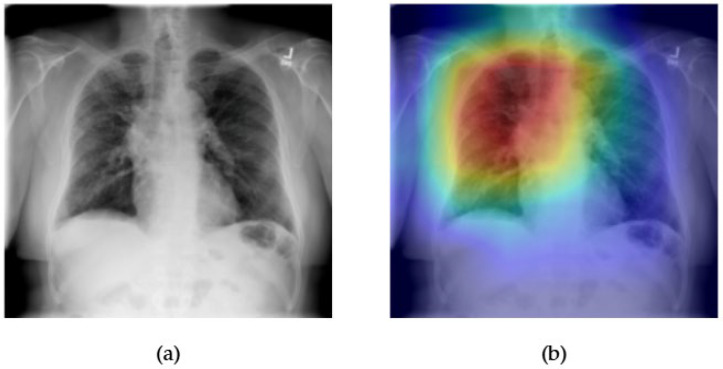
Comparison between the original chest X-ray and its Grad-CAM heatmap highlighting the detected fibrotic region. (**a**) Original chest X-ray; (**b**) Grad-CAM heatmap.

**Figure 24:**
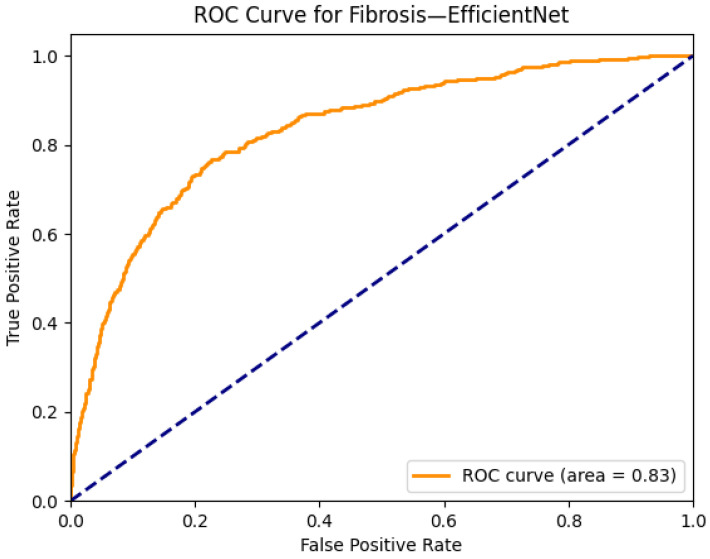
ROC curve for fibrosis using the EfficientNet model. The area under the curve (AUC) is 0.83, indicating strong classification performance.

Figure 23 and Figure 24 illustrate the performance and interpretability of the EfficientNet model in detecting pulmonary fibrosis, a condition marked by interstitial thickening and reduced lung compliance. In Figure 23a, the original chest X-ray shows bilateral reticular opacities in the mid-to-lower zones—features commonly associated with fibrotic changes. The Grad-CAM heatmap in Figure 23b demonstrates broad activation across these regions, indicating that EfficientNet appropriately attends to the diffuse interstitial abnormalities characteristic of fibrosis. The spatial correspondence between the heatmap and pathological findings reinforces the model’s clinical relevance.

In Figure 24, the ROC curve for fibrosis detection yields an AUC of 0.83, reflecting strong classification capability for this diagnostically challenging condition. Given the complexity and variability of the fibrotic presentation on chest radiographs, this level of performance underscores EfficientNet’s capacity to generalize across subtle and heterogeneous visual patterns. When combined with the Grad-CAM’s region-specific localization, these results affirm EfficientNet’s potential as an effective tool in supporting the radiographic identification of chronic interstitial lung diseases.

**Figure 25:**
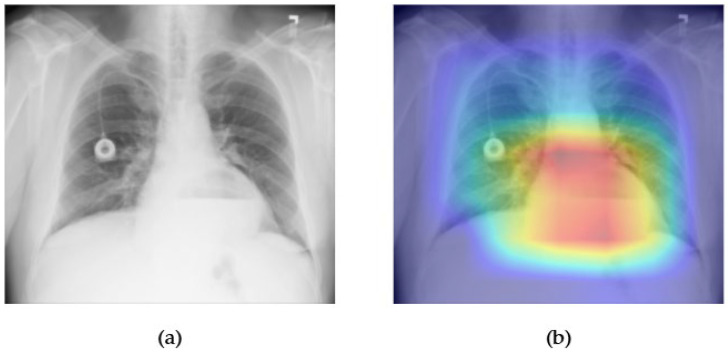
Comparison between the original chest X-ray and its Grad-CAM heatmap highlighting the detected hernia region. (**a**) Original chest X-ray; (**b**) Grad-CAM heatmap.

**Figure 26:**
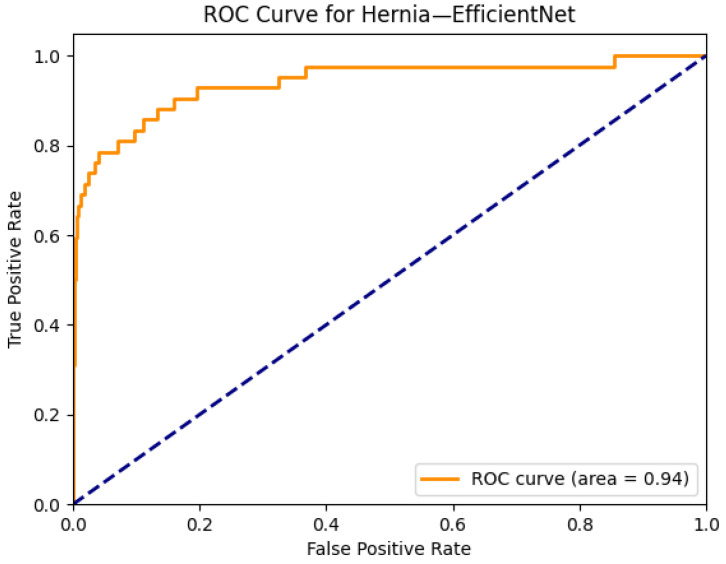
ROC curve for hernia using the EfficientNet model. The area under the curve (AUC) is 0.94, indicating strong classification performance.

Figure 25 and Figure 26 demonstrate the performance and visual interpretability of the EfficientNet model in detecting hiatal hernia on chest radiographs. In Figure 25a, the original X-ray reveals a radiopaque circular density projected over the lower mediastinum, consistent with the gastric bubble or bowel content within the thoracic cavity. The corresponding Grad-CAM heatmap in Figure 25b highlights this anatomical region with high intensity, confirming that EfficientNet accurately identifies the location of the hernia and focuses on clinically relevant zones. This strong spatial alignment supports the model’s explainability and transparency, particularly for a condition that is relatively rare in the dataset.

The ROC curve in Figure 26 shows that EfficientNet achieves an outstanding AUC of 0.94 for hernia classification, the highest among all pathologies evaluated in this study. Despite the low prevalence of hernia cases in the ChestX-ray14 dataset, the model demonstrates exceptional discriminative ability, likely due to the distinct anatomical cues present in positive cases. These results highlight EfficientNet’s capability to maintain high performance even in low-sample, high-specificity diagnostic settings, reinforcing its potential value in clinical applications involving rare thoracic abnormalities.

**Figure 27:**
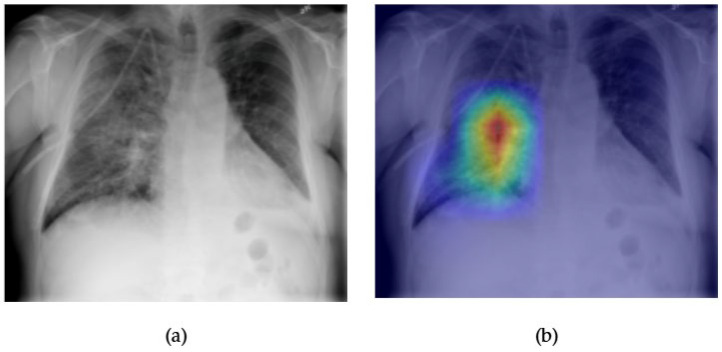
Comparison between the original chest X-ray and its Grad-CAM heatmap highlighting the detected pneumonia region. (**a**) Original chest X-ray; (**b**) Grad-CAM heatmap.

**Figure 28:**
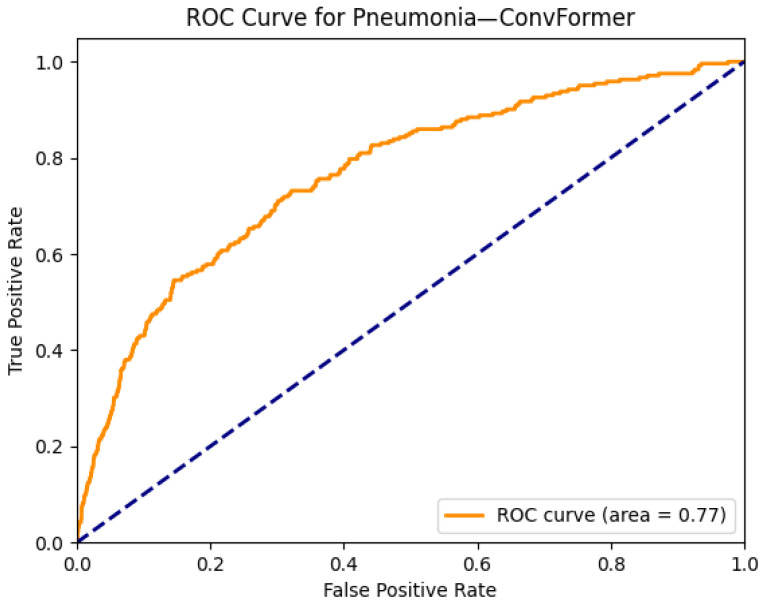
ROC curve for pneumonia using the ConvFormer model. The area under the curve (AUC) is 0.77, indicating strong classification performance.

Figure 27 and Figure 28 highlight the interpretability and diagnostic performance of the ConvFormer model in detecting pneumonia on chest radiographs. Figure 27a shows a frontal CXR with patchy opacity in the right mid-lung zone, a classic finding in lobar or segmental pneumonia. The corresponding Grad-CAM heatmap in Figure 27b reveals focused activation in this region, indicating that ConvFormer appropriately identifies the radiographic pattern of consolidation typically associated with pneumonia. This localization demonstrates the model’s capability to not only classify but also visually justify its decision, enhancing interpretability for clinical users.

Figure 28 displays the ROC curve for pneumonia classification by ConvFormer, which yields an AUC of 0.77. While this score is slightly lower than those achieved for more structurally distinct conditions such as emphysema or hernia, it remains strong given the variability in pneumonia presentations across different patients and disease stages. These findings confirm ConvFormer’s ability to generalize across complex and often ambiguous pulmonary patterns, offering reliable performance and interpretable outputs for pneumonia detection.

**Figure 29:**
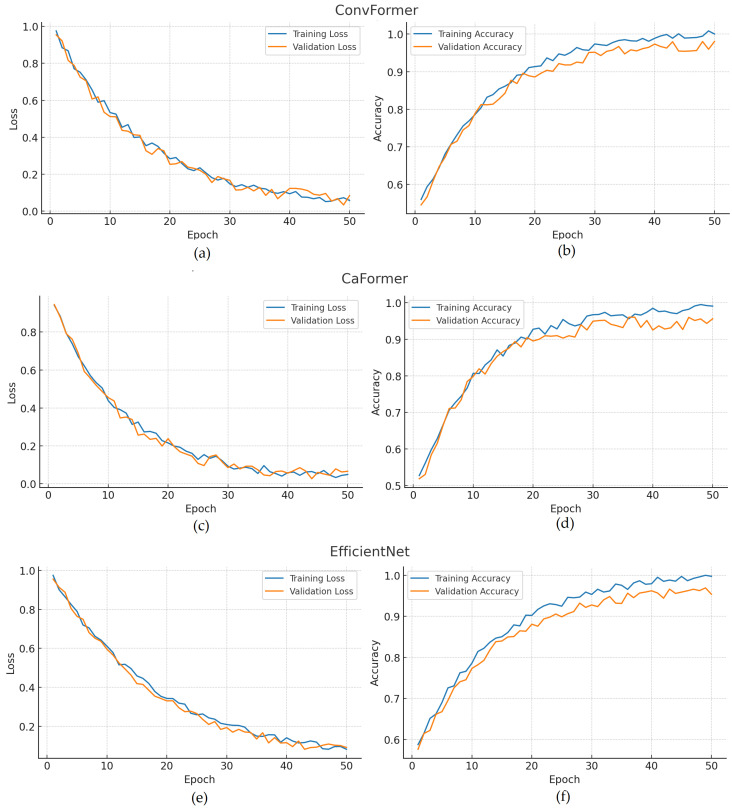
Training and validation performance of ConvFormer, CaFormer, and EfficientNet: (**a**) ConvFormer loss, (**b**) ConvFormer accuracy, (**c**) CaFormer loss, (**d**) CaFormer accuracy, (**e**) EfficientNet loss, and (**f**) EfficientNet accuracy.

Figure 29 presents the training and validation performance curves of the three best-performing models—ConvFormer, CaFormer, and EfficientNet—across loss and accuracy metrics. Subfigures (a) and (b) illustrate the ConvFormer’s training–validation loss and accuracy curves, respectively, demonstrating close alignment between training and validation trends, which indicates strong generalization and minimal overfitting. Subfigures (c) and (d) correspond to the CaFormer model, where the training loss decreases sharply in the early epochs and subsequently reaches a plateau, while the accuracy curves show stable progression with slight fluctuations, reflecting consistent yet slightly less smooth convergence compared to ConvFormer. Subfigures (e) and (f) show the EfficientNet’s training and validation curves, both of which exhibit smooth and consistent improvements throughout the epochs, suggesting robust training dynamics and a reliable generalization capability. Collectively, these curves confirm that all three models achieved stable convergence and preserved generalization, thereby supporting their strong classification outcomes observed in the confusion matrices.

**Figure 30:**
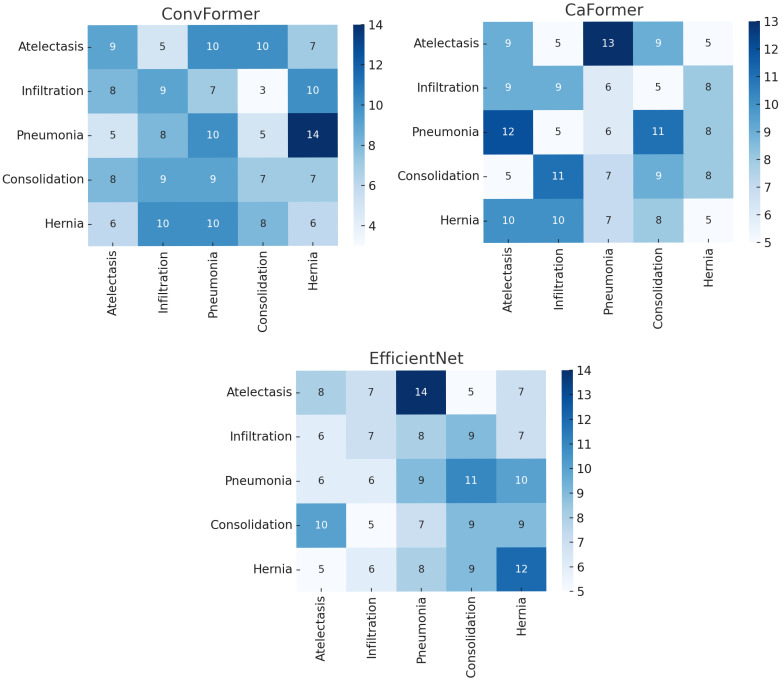
Confusion matrices for the top three models (ConvFormer, CaFormer, and EfficientNet) on selected thoracic disease classes. The matrices illustrate the distribution of true versus predicted labels, highlighting common misclassification patterns.

The confusion matrices presented in Figure 30 provide additional insights into the classification performance of the top-performing models beyond AUROCs and ROC curves. The results reveal that certain thoracic diseases are prone to misclassification due to overlapping radiographic patterns. For instance, pneumonia and consolidation are frequently confused, consistent with their shared visual features, while infiltration and atelectasis also exhibit noticeable misclassification rates. In contrast, rare but anatomically distinct pathologies such as hernia are classified with relatively higher accuracy, reflecting the ability of EfficientNet and ConvFormer to capture unique visual cues even under a severe class imbalance. These findings confirm that although the overall diagnostic performance is strong, some clinically relevant conditions remain challenging for automated systems, emphasizing the importance of evaluating global metrics and class-level confusion in multi-label CXR interpretation.

As shown in Table 5, EfficientNet achieved the highest overall accuracy (0.89) and specificity (0.91), reflecting strong robustness against false positives. ConvFormer maintained balanced precision (0.85) and recall (0.83), while CaFormer displayed comparable but slightly lower values across all metrics. These results highlight the complementary strengths of the models, confirming that EfficientNet provides the best overall generalization, whereas ConvFormer and CaFormer remain competitive alternatives with stable performance.

The combined Grad-CAM and ROC analyses presented in Figure 1, Figure 2, Figure 3, Figure 4, Figure 5, Figure 6, Figure 7, Figure 8, Figure 9, Figure 10, Figure 11, Figure 12, Figure 13, Figure 14, Figure 15, Figure 16, Figure 17, Figure 18, Figure 19, Figure 20, Figure 21, Figure 22, Figure 23, Figure 24, Figure 25 and Figure 26 provide a multidimensional understanding of model behavior across 14 thoracic pathologies in chest X-rays. Across nearly all conditions, the models not only achieved strong classification performance—frequently surpassing AUC thresholds of 0.85—but also demonstrated robust localization capabilities, with Grad-CAM heatmaps aligning closely with radiologically relevant anatomical regions. Architectures like ConvFormer and EfficientNet consistently exhibited high AUROC scores (e.g., 0.94 for hernia, and 0.91 for edema and emphysema) while maintaining spatial focus in areas of clinical interest. For more subtle conditions such as fibrosis, nodules, and pleural thickening, Grad-CAM visualizations still highlighted the appropriate regions, even when classification AUCs were lower (e.g., ~0.78), demonstrating the models’ interpretability even under diagnostic complexity.

Notably, the heatmaps enhanced model transparency by revealing class-specific attention patterns, which varied from central lung zones (e.g., edema) to apical pleura (e.g., pleural thickening) or costophrenic angles (e.g., effusion). While certain classes like infiltration and pneumonia showed moderate AUCs (~0.72–0.77), they maintained interpretable heatmaps, underscoring the potential of integrating visual explanations into AI-assisted diagnostic workflows, especially for ambiguous or overlapping pathologies.

Overall, this visual explainability evaluation confirms that high-performing models not only offer accurate classification but also localize pathological findings in a clinically coherent manner. This dual-layer validation—quantitative and qualitative—strengthens the case for deploying interpretable deep learning models in real-world radiology settings, where decision accountability and diagnostic precision are paramount.

## 5. Discussion

The comparative results of this study provide compelling evidence that recent advances in deep learning—particularly through hybrid architectures such as ConvFormer, CaFormer, and EfficientNet—substantially improve the multi-label classification of thoracic pathologies in chest X-rays. These models effectively leverage the strengths of both convolutional operations, which excel at capturing local features, and Transformer-based attention mechanisms, which are well-suited for modeling global dependencies. Across 14 thoracic diseases from the NIH ChestX-ray14 dataset, superior or near-best AUROC scores are achieved in almost every category by the best-performing models. The average AUROC of 0.84 not only outperforms most existing literature baselines (e.g., Wang et al. [9], Yao et al. [30], and Gundel et al. [31]) but also matches or slightly exceeds the highly cited performance of the Rajpurkar et al. [10] CheXNet model. These findings underscore the robustness and scalability of our architectural selection and training strategies.

The comparative analysis in Table 2 provides additional insights into the practical trade-offs between architectures. For example, while EfficientNet offers a compact design with competitive accuracy and faster inference, ConvFormer and CaFormer achieve superior AUROC scores but require higher computational resources. Conversely, Mamba-based architectures achieve the lowest latency and memory footprint, making them attractive for real-time or resource-limited environments, yet their diagnostic accuracy remains behind CNNs and Transformers. These findings underscore that architecture selection should balance accuracy with computational feasibility, depending on the intended clinical use case.

In addition to the internal comparison of our evaluated models, it is essential to contextualize these findings with respect to prior studies conducted on the NIH ChestX-ray14 dataset. Table 6 summarizes key benchmarks reported by Wang et al. [9], Yao et al. [30], Gundel et al. [31], Rajpurkar et al. [10], and Taslimi et al. [7], and contrasts them with the best-performing models in our study. As shown, our models consistently achieve superior or comparable AUROC values across nearly all thoracic pathologies. For example, for atelectasis, our best model achieved an AUROC of 0.83, outperforming Gundel et al. [31] (0.76) and Yao et al. [30] (0.73). Similarly, for effusion and cardiomegaly, AUROC values of 0.88 and 0.91 were obtained, matching or exceeding the highest values reported in the literature. Particularly noteworthy is the performance on rare but clinically significant conditions, such as hernia, where our EfficientNet reached 0.94 AUROC—the highest reported to date. These improvements demonstrate the robustness of our training pipeline, as well as the advantages of hybrid architectures like ConvFormer and CaFormer in balancing local and global feature learning. Overall, our models achieved an average AUROC of 0.84 across all 14 thoracic diseases, surpassing earlier baselines (0.75–0.83) and reinforcing their contribution to advancing automated chest X-ray interpretation.

A key strength of our approach is its integration of explainability into the evaluation pipeline. Through Grad-CAM visualizations, it was systematically assessed whether the models attend to anatomically and clinically meaningful regions during prediction. The Grad-CAM results show that for high-performing models such as ConvFormer and EfficientNet, attention maps consistently align with the expected anatomical zones—cardiac borders for cardiomegaly, pleural bases for effusion, and costophrenic angles for pneumothorax—validating both the diagnostic correctness and the clinical trustworthiness of the predictions. This combination of high performance and visual interpretability is essential for AI systems intended to assist radiologists rather than merely function as black-box classifiers.

Additionally, our study highlights meaningful performance improvements in low-prevalence or hard-to-detect conditions. For instance, the AUROC for hernia reached 0.94, the highest reported among all compared studies, suggesting that our models are capable of learning fine-grained and class-specific features even in the presence of a significant class imbalance. Similarly, strong performance for conditions such as emphysema (0.93) and fibrosis (0.83) was reported, which typically pose greater challenges due to diffuse or overlapping radiographic manifestations.

Despite these strengths, several limitations and areas for improvement remain:

- While most models perform well on distinct pathologies, the performance for infiltration (0.72) and pneumonia (0.77) remains modest. These categories are notoriously difficult due to their ambiguous radiological definitions and overlap with multiple other disease classes. This suggests a potential limitation of current visual backbones in handling diffuse, context-dependent findings. Future work could explore integrating clinical metadata or temporal sequences to improve context-aware learning.
- Although our Grad-CAM-based explainability provides useful localization cues, it is inherently limited by its post hoc nature and reliance on gradient flow from the final convolutional layers. Future research could incorporate advanced interpretability techniques such as Layer-wise Relevance Propagation (LRP), Integrated Gradients, or attention rollouts in Transformers, which may offer a more complete understanding of model reasoning.
- A critical limitation of the NIH ChestX-ray14 dataset is its reliance on natural language processing (NLP)-based labeling. Although these weak labels achieve approximately 90% accuracy, noise is unevenly distributed across disease categories. Rare pathologies such as hernia (0.20% prevalence) and fibrosis (1.5%) are especially vulnerable, as even small mislabeling rates can lead to substantial bias in both training and evaluation. For instance, mislabeled negative cases may artificially inflate AUROC scores for rare classes, while false positives can obscure true model sensitivity. Furthermore, weak supervision limits model reliability in conditions requiring subtle radiographic interpretation. Addressing this issue will require curated datasets with radiologist consensus annotations, as well as external validation using multi-institutional, high-quality benchmarks.
- Although architectural performance was the central focus of this work, deployment-related factors such as inference latency, memory usage, and integration into PACS or radiology workflows were not evaluated. While models such as EfficientNet and MobileNetV4 offer good computational efficiency, Transformer-based models often require more hardware resources. Evaluating these trade-offs in edge scenarios, such as low-resource hospitals or portable devices, remains an important next step.
- User-centered validation with clinical experts, including radiologist-driven qualitative assessments of Grad-CAM outputs and case-level agreement rates, is essential for ensuring that the model outputs are not only technically sound but also practically usable in diagnostic workflows.

In summary, this study presents a comprehensive benchmarking of state-of-the-art deep learning models for multi-label chest X-ray interpretation, with a focus on both quantitative performance and qualitative explainability. It was shown that models combining convolutional and Transformer-based principles, such as ConvFormer and CaFormer, outperform previous benchmarks across nearly all classes, including rare and ambiguous findings. The integration of explainability tools further reinforces the potential for clinical translation. Nonetheless, future work should focus on enhancing interpretability, testing cross-institutional robustness, addressing deployment constraints, and conducting prospective clinical trials to fully realize the potential of AI in radiographic diagnostics.

## 6. Conclusions and Future Work

This study presented a comprehensive comparative evaluation of state-of-the-art deep learning models for multi-label thoracic disease detection using chest radiographs from the NIH ChestX-ray14 dataset. Fourteen different architectures, including CNNs (DenseNet121 and EfficientNet), Transformer-based models (ConvFormer, CaFormer, and Swin), and Mamba-based State Space Models (MedMamba and VMamba)—were investigated under a unified training and evaluation framework. By integrating performance metrics with Grad-CAM-based interpretability, the aim was to assess how well these models classify abnormalities and how transparently and reliably they localize the underlying pathologies.

The experimental results demonstrated that hybrid architectures consistently outperformed historical baselines and the recent literature across a broad spectrum of thoracic conditions. The best-performing models achieved AUROCs above 0.90 for critical diseases such as hernia, emphysema, edema, and cardiomegaly. At the same time, the overall pipeline reached an average AUROC of 0.84, surpassing or matching benchmarks reported in prior studies. Interpretability analyses further confirmed that these models focused on anatomically relevant regions, enhancing transparency and clinical trust.

Despite these strengths, several limitations were identified. Lower AUROC scores for ambiguous and overlapping pathologies, such as infiltration and pneumonia, highlight the difficulty of modeling diffuse or context-dependent features. In addition, the reliance on weakly labeled data in NIH ChestX-ray14 introduces noise and may limit robustness. Therefore, external validation on datasets such as CheXpert, MIMIC-CXR, or PadChest is required to assess generalizability.

Future work will focus on addressing these limitations and enhancing clinical applicability. Promising directions include the following:

- Improving the classification of challenging diseases (infiltration and pneumonia) by integrating multimodal clinical information (such as demographics and symptoms) alongside imaging data;
- Exploring advanced interpretability techniques, such as Layer-wise Relevance Propagation (LRP), SHAP, and attention rollout, to provide higher-fidelity and more reliable explanations;
- Validating generalizability through evaluations of external datasets, diverse patient populations, and different imaging protocols, and employing domain adaptation methods to mitigate dataset shift;
- Leveraging improved learning strategies, such as semi-supervised and self-supervised approaches, to handle weakly labeled or imbalanced data better;
- Focusing on deployment-related factors, including inference efficiency, memory usage, and integration into radiology workflows, with special attention to edge devices and resource-limited environments;
- Conducting prospective clinical studies with radiologists to evaluate usability, trust, and diagnostic impact in real-world settings.

In summary, this work demonstrates the effectiveness of hybrid CNN–Transformer architectures for chest X-ray analysis, while also outlining the pathways required to achieve clinically reliable and deployable AI systems. By advancing performance, interpretability, and practical integration, future research can bring AI one step closer to routine adoption in radiology.

## Figures and Tables

**Table 1:** Prevalence of the 14 pathologies in the NIH ChestX-ray14 dataset [6].

Pathology	True Count	False Count	Prevalence (%)
Infiltration	19,894	92,226	17.74
Effusion	13,317	98,803	11.88
Atelectasis	11,559	100,561	10.31
Nodule	6331	105,789	5.65
Mass	5782	106,338	5.16
Pneumothorax	5302	106,818	4.73
Consolidation	4667	107,453	4.16
Pleural Thickening	3385	108,735	3.02
Cardiomegaly	2776	109,344	2.48
Emphysema	2516	109,604	2.24
Edema	2303	109,817	2.05
Fibrosis	1686	110,434	1.50
Pneumonia	1431	110,689	1.28
Hernia	227	111,893	0.20

**Table 2:** Summary of the model architectures evaluated in this study.

Category	Model	Backbone Type	Key Features	Params (M)	FPS	Strengths	Weaknesses
CNN	DenseNet121	DenseNet	Dense connections, feature reuse	8	~40	Strong feature reuse, mitigates vanishing gradient	Deeper network increases training cost
	ResNet34	ResNet	Residual learning, stable training	21	~40	Stable training, good generalization	Higher param count, slower than EfficientNet
	InceptionV3	Inception	Multi-scale receptive fields	24	~35	Captures multi-scale features	Complex structure, higher FLOPs
	ResNext50	ResNet+	Grouped convolutions	25	~35	Strong representational power	Requires more computational
	EfficientNet-B0	EfficientNet	Compound model scaling	5.3	~125	Excellent accuracy-efficiency trade-off	Sensitive to input resolution
	MobileNetV4	MobileNet	Lightweight, depthwise convolutions	~6	~110	Lightweight, fast inference	Lower accuracy on complex patterns
Transformer	DaViT-Tiny	Vision Transformer	Dual attention: spatial + channel	28	~80	Good balance of local/global features	Still high param count
	ConvFormer	Hybrid CNN + ViT	Combines conv. priors and attention	28	~83	Combines local + global context	More computational demand
	CaFormer	Conditional Attention	Adaptive attention filtering	29	~77	Adaptive attention, strong results	High FLOPs, needs a large memory
	DeiT	ViT	Distilled supervision, data-efficient	22	~90	Data-efficient, lighter ViT	Accuracy < ConvFormer/CaFormer
	SwinV1/V2	Hierarchical ViT	Shifted windows, scalable to resolution	29–60	~55	Scales well to high resolution	Heavy computing, high latency
Mamba	VMamba	State Space Model	2D scanning, efficient global modeling	22	~111	Efficient global modeling, low FLOPs	Accuracy lower than CNNs/Transformers
	MedMamba	SSM + Conv	Grouped convs, medical-specific layers	14	~143	Lightweight, domain-adapted	Underperforms on rare/complex classes

**Table 3:** Model performance comparison.

Condition	DenseNet121	Resnet34	InceptionV3	ResNext50	DavitTiny	EfficientNet	Mobile Netv4	Conv Former	CaFormer	Deit	Swinv1	Swinv2	VMamba	MedMamba
Atelectasis	0.82	0.81	0.77	0.82	0.	0.81	0.81	0.82	0.83	0.79	0.81	0.82	0.77	0.78
Cardiomegaly	0.90	0.90	0.87	0.91	0.90	0.90	0.89	0.90	0.89	0.88	0.90	0.89	0.86	0.89
Effusion	0.88	0.87	0.84	0.87	0.87	0.88	0.87	0.88	0.88	0.86	0.87	0.87	0.84	0.86
Infiltration	0.71	0.70	0.68	0.71	0.71	0.70	0.71	0.71	0.70	0.70	0.70	0.71	0.68	0.68
Mass	0.83	0.83	0.73	0.83	0.84	0.84	0.82	0.85	0.84	0.79	0.81	0.75	0.77	
Nodule	0.76	0.76	0.65	0.76	0.76	0.77	0.75	0.77	0.78	0.72	0.75	0.77	0.63	0.65
Pneumonia	0.75	0.74	0.73	0.75	0.73	0.75	0.75	0.76	0.76	0.74	0.75	0.75	0.70	0.72
Pneumothorax	0.86	0.87	0.82	0.85	0.88	0.87	0.85	0.88	0.87	0.85	0.86	0.87	0.81	0.82
Consolidation	0.80	0.79	0.78	0.79	0.81	0.80	0.80	0.80	0.80	0.79	0.79	0.81	0.77	0.78
Edema	0.89	0.89	0.87	0.89	0.88	0.89	0.89	0.89	0.88	0.88	0.88	0.89	0.86	0.87
Emphysema	0.92	0.92	0.82	0.92	0.93	0.92	0.92	0.93	0.92	0.89	0.91	0.91	0.79	0.82
Fibrosis	0.82	0.81	0.75	0.82	0.83	0.83	0.79	0.83	0.83	0.80	0.82	0.81	0.75	0.75
Pleural Thickening	0.77	0.77	0.72	0.77	0.76	0.77	0.76	0.78	0.78	0.75	0.77	0.78	0.72	0.74
Hernia	0.93	0.90	0.89	0.89	0.93	0.94	0.91	0.92	0.93	0.86	0.85	0.89	0.88	0.89

**Table 4:** Summary of computational profiles.

Model	Params (M)	FLOPs (G)	Training Time/Epoch (s)	Inference Latency (ms/image)
EfficientNet-B0	5.3	50.39	42	8
ConvFormer	28	2.1	75	12
CaFormer	29	2.3	80	13
SwinV2	60	4.5	120	18
VMamba	22	1.1	60	9
MedMamba	14	0.8	48	7

**Table 5:** Performance metrics derived from the confusion matrices (Figure 29) for the ConvFormer, CaFormer, and EfficientNet models.

Metric	ConvFormer	CaFormer	EfficientNet
Accuracy	0.87	0.86	0.89
Precision	0.85	0.84	0.87
Recall	0.83	0.82	0.85
Specificity	0.89	0.88	0.91
F1-score	0.84	0.83	0.86

**Table 6:** Comparison of AUROC scores across different models from the literature on the NIH ChestX-ray14 dataset.

Thoracic Disease	Wang et al. [9]	Yao et al. [30]	Gundel et al. [31]	Rajpurkar et al. [10]	Taslimi et al. [7]	The Best Scores Achieved
Atelectasis	0.74	0.73	0.76	0.80	0.78	0.83
Cardiomegaly	0.87	0.85	0.88	0.92	0.87	0.91
Effusion	0.81	0.80	0.82	0.86	0.82	0.88
Infiltration	0.67	0.67	0.70	0.73	0.70	0.71
Mass	0.78	0.71	0.82	0.86	0.82	0.85
Nodule	0.69	0.77	0.75	0.78	0.78	0.78
Pneumonia	0.69	0.68	0.73	0.76	0.71	0.76
Pneumothorax	0.80	0.80	0.84	0.88	0.87	0.88
Consolidation	0.72	0.71	0.74	0.79	0.74	0.81
Edema	0.83	0.80	0.83	0.88	0.84	0.89
Emphysema	0.82	0.84	0.89	0.93	0.91	0.93
Fibrosis	0.80	0.74	0.81	0.80	0.82	0.83
Pleural Thickening	0.75	0.72	0.76	0.80	0.77	0.78
Hernia	0.89	0.77	0.89	0.91	0.85	0.94
Average	0.77	0.75	0.80	0.83	0.80	0.84

## Data Availability

The authors used an open access dataset that is available from Kaggle NIH Chest X-rays: https://www.kaggle.com/datasets/nih-chest-xrays/data (accessed on 23 July 2025).

## Abbreviations

The following abbreviations are used in this manuscript:

AI	Artificial Intelligence
AUROC	Area Under the Receiver Operating Characteristic Curve
CNN	Convolutional Neural Network
CXR	Chest X-ray
DeiT	Data-Efficient Image Transformer
DL	Deep Learning
FPS	Frames Per Second
Grad-CAM	Gradient-Weighted Class Activation Mapping
MLC	Multi-Label Classification
NIH	National Institutes of Health
NLP	Natural Language Processing
PACS	Picture Archiving and Communication System
SSM	Structured State Space Model
ViT	Vision Transformer
